# Lead Causes Lipid Droplet Accumulation by Impairing Lysosomal Function and Autophagic Flux in Testicular Sertoli Cells

**DOI:** 10.3390/toxics13030175

**Published:** 2025-02-28

**Authors:** Chengwei Guo, Lingqiao Wang, Ke Cui, Guowei Zhang, Yao Tan, Weiyan Chen, Yiqi Wang, Jijun Liu, Wenbin Liu, Guanghui Zhang, Ziyuan Zhou

**Affiliations:** 1 Department of Environmental Health, College of Preventive Medicine, Third Military Medical University (Army Medical University), Chongqing 400038, China; 15123001480@163.com (C.G.); mamababa520qq@163.com (L.W.); cuike@tmmu.edu.cn (K.C.); zhangguowei@tmmu.edu.cn (G.Z.); xiaoyue7122@tmmu.edu.cn (Y.T.); weiyanchen@tmmu.edu.cn (W.C.); wangyiqi@tmmu.edu.cn (Y.W.); liuwenbin@tmmu.edu.cn (W.L.); zhgh221@tmmu.edu.cn (G.Z.); 2 Chongqing Center for Disease Control and Prevention, Chongqing 400707, China; cqliujijun2007@163.com

**Keywords:** autophagy, lead, male reproduction, TFE3, ROS

## Abstract

Lead (Pb) is one of the most common environmental pollutants that negatively impacts male reproductive health. Thus far, the underlying molecular mechanisms of Pb-induced reproductive toxicity are still not well understood. In this study, 64 male ICR mice were given drinking water with Pb (0, 100, 200, and 300 mg/L) for 90 days. We found that exposure to 300 mg/L Pb resulted in reduced sperm quality and elevated autophagy-related protein levels in the mouse testes. Our findings indicate that the Pb hindered the autophagic clearance by impairing the lysosomes’ function and then obstructing the fusion of lysosomes and autophagosomes. The autophagy cycle obstruction prevented the lipid droplets from breakdown and led to their accumulation in the Sertoli cells. In turn, the ccytotoxic effects that resulted from the interruption of the autophagy maturation stage, instead of the elongation phase, could be alleviated by either Chloroquine or Bafilomycin A1. Furthermore, exposure to 400 μM Pb initiated the TFE3 nuclear translocation and caused the increased expression of its target genes. Then, the knockdown of TFE3 reduced the formation of the autophagosome. In addition, the use of the antioxidant NAC notably enhanced the autophagic activity and reduced the occurrence of lipid droplets in the Sertoli cells. This study demonstrated that Pb disrupted the autophagic flow, which caused lipid droplet accumulation in the TM4 cells. Consequently, focusing on the maturation stage of autophagy might offer a potential therapeutic approach to alleviate male reproductive toxicity caused by Pb exposure.

## 1. Introduction

Lead (Pb) is widely used as an industrial raw material in metallurgy, batteries, printing, and the chemical and other industries, causing considerable pollution to the natural environment and human diet [1]. Additionally, Pb toxicity is amplified by its prolonged half-life. The general population exposure to Pb mainly occurs through contaminated food, drinking water, leaded gasoline, and dust [2]. Considering the amplified toxicity derived from Pb’s non-degradability and long biological half-life in both the environment and organisms, there has been growing concern that occupational or environmental Pb exposure may affect male reproductive function. Pb is recognized to possess male reproductive toxicity linking to growth retardation, pubertal maturation delay, and semen quality decline [3]. Further research also demonstrated that Pb has harmful impacts on male reproductive health by disrupting hormone regulation and spermatogenesis [4,5]. However, the mechanisms underlying Pb-induced reproductive toxicity still remain unclear.

Sertoli cells, which serve as a physical barrier and supply nutrients to developing germ cells, are the primary targets of Pb toxicity. Studies have reported that Pb induces pyroptosis in Sertoli cells, which, in turn, leads to testicular fibrosis [6]. Earlier research demonstrated that exposure to 10 μM Pb for 24 h resulted in a significant increase in reactive oxygen species [7]. Sertoli cells absorb glucose from the extracellular environment and transform it into lactate, supplying energy to germ cells [8]. In this metabolic scenario, the oxidation of fatty acids stored in lipid droplets generates the ATP required by the Sertoli cells [9]. It is reported that a 1 μM Pb treatment for 24 h can affect lactate production and transportation in Sertoli cells [10]. In addition, lipid droplets are formed when Sertoli cells engulf and metabolize dead germ cells [11]. The dysfunction of lysosomes and the buildup of lipids have been documented in the testicular tissue of individuals suffering from late onset hypogonadism [12]. After rats were treated with Pb water at a dose of 25 mg/kg b.w. for 90 days, an increased lysosome content and phagocytosis of apoptotic bodies were also observed in the Sertoli cells [13]. The above research demonstrated that Pb could negatively influence the lysosomal activity of support cells, leading to irregularities in lipid metabolism and phagocytic processes.

Autophagy refers to a mechanism through which eukaryotic cells utilize lysosomes to break down their own cytoplasmic proteins and deteriorated organelles, which is regulated by genes associated with autophagy [14]. This mechanism can control the levels of lipids within the cell by decomposing lipid droplets, which is known as a process of lipophagy [15]. Autophagy plays an important role in the normal growth and development of male reproductive organs but also in male reproductive physiological processes, including blood–testis barrier integrity maintenance, testosterone synthesis, spermatogenesis, testosterone synthesis, and the stability of the blood–testis barrier [16,17]. In male reproductive toxicity caused by environmental endocrine-disrupting chemicals (EDCs), autophagy is also two-sided, with both protective and damaging effects [18,19]. Nevertheless, the function of autophagy in Pb-induced toxicity affecting testicular organs is still unclear, necessitating further investigation into its mechanisms. The protein TFE3, which contains a bHLH-leucine zipper domain, has been recognized as a key regulator for gene expression associated with autophagy and the formation of lysosomes [20]. Thus, in each stage of autophagy, TFE3 is involved through promoting the formation of the autophagosome and aiding its fusion with lysosomes, as well as maintaining the degradation function of lysosomes. However, more details still need to be elucidated to what extent does abnormal autophagy contribute to Pb-induced male reproductive toxicity and how autophagy is regulated by TFE3.

In the current research, we explored the effects of a 90-day Pb exposure on sperm quality and testicular autophagy-related proteins in mice. Furthermore, we investigated the particular elements of the impact of autophagy on the Pb-triggered cytotoxicity and its underlying upstream mechanism in TM4 cells. To explain the relationship between the observed impairment of sperm motility in mice and the markedly increased expression of autophagy-related proteins in the testes, we further evaluated the effects of Pb on various stages of autophagy and the role of TFE3 in regulating the Pb-triggered autophagy. To our knowledge, this research first demonstrated that Pb exposure led to lipid droplet accumulation in Sertoli cells, and antioxidant NAC significantly mitigated this process.

## 2. Materials and Methods

### 2.1. Animals and Treatment

A total of 64 five-week-old male SPF ICR mice (Vital River Laboratory Animal Technology Co., Beijing, China) were housed with ad libitum access to water and food, with five per cage. A regular light and dark cycle was maintained for 12 h each. The male mice were randomly divided into four groups according to body weight and given distilled water with lead acetate Pb (AC)_2_ concentrations of 0, 100, 200, and 300 (mg/L, as Pb^2+^). The Pb treatments were administered for a duration of 90 days. All animal procedures complied with the National Institute of Health’s ethical guidelines for laboratory animals and were approved by the Laboratory Animal Welfare and Ethics Committee of the Army Medical University (AMUWEC 20230252).

### 2.2. Semen Quality Analysis

The computer-assisted sperm analysis system (CASA; Microptic S.L., Barcelona, Spain) was employed to evaluate the parameters of sperm quality, including sperm concentration, total count, progressive mobility, and total motility.

### 2.3. Histological Analysis and Immunofluorescent Labeling in Murine Testicular Tissue

Mice were sacrificed after anesthesia with pentobarbital and testes were fixed in 4% paraformaldehyde overnight. For the HE staining, we embedded the testes in paraffin according to standard protocols [21]. A 5 μm thick tissue section was prepared from the central region, stained with hematoxylin–eosin, and examined using a light microscope. For immunofluorescence, the paraffin sections were fixed, permeabilized, and subjected to blocking using standardized protocols. The slides were incubated at 4 °C overnight with rabbit anti-LC3 (1:500, CST, 43566S, Danvers, MA, USA) and rabbit anti-P62 (1:500, CST, D6M5X) anti-bodies in immunostaining dilution buffer, then incubated with Donkey Anti-Rabbit IgG (H+L) conjugated with Alexa Fluor 555 (Beyotime, Shanghai, China) at a 1:200 dilution for one hour at 37 °C, ensuring protection from light. The nucleus was stained for 5 min with DAPI staining solution (Beyotime, China) to identify the labels. The stained specimens were analyzed with a Lecia confocal laser scanning microscope (Lecia, Stellaris 5).

### 2.4. Cell Culture and Treatment

A Sertoli cell line from mouse testicles (TM4) was purchased from Pricella (Wuhan, China) and was originally sourced from the American Type Culture Collection (ATCC). The cells were cultured in DMEM/F12 medium (Thermo Scientific, MA, USA) supplemented with 10% fetal calf serum and 1% penicillin/streptomycin in a 37 °C humidified incubator with a 5% CO_2_ atmosphere. When the cells reached about 70% confluence, they were exposed to various concentrations (0, 25, 100, and 400 μM) of Pb for either 24 h or 48 h. Lead acetate (Sigma, 32307, St. Louis, MS, USA) was dissolved in ultrapure water and diluted in DMEM/F12 medium.

### 2.5. Cell Viability

Rapamycin (TargetMol, T1537, Boston, MA, USA), 3-Methyladenine (TargetMol, T1879), chloroquine (TargetMol, T8689), and bafilomycin A1 (TargetMol, T6740) were dissolved in DMSO, and N-Acetylcysteine (TargetMol, T0875) was dissolved in ultrapure water. The TM4 cells were exposed to rapamycin (R, 200 nM), 3-Methyladenine (3-MA, 2 mM), chloroquine (CQ, 20 µM), bafilomycin A1 (Baf, 10 nM), or N-Acetylcysteine (NAC, 2 mM), either alone or in combination with Pb (400 µM), for a duration of 24 h. The working concentrations of these reagents were derived from previous reports [22]. The cell viability was measured by the MTT kit (Solarbio, M1020, Beijing, China) to assess the cytotoxic effects of Pb on the TM4 cell lines [23]. In brief, 5000 cells were inoculated into a 96-well plate and cultured for 24 h. After 24 h of Pb treatment, 10 μL of MTT solution was added to each well and incubated for 4 h. The absorbance at 490 nm was measured by an enzyme labeling instrument (Molecular Devices, M3, San Jose, CA, USA). The viability of the cells was represented as the ratio of absorbance from treated cultures in comparison with control cultures.

### 2.6. Transmission Electron Microscopy

In order to investigate the autophagy triggered by Pb on the TM4 cells, the treated cells underwent washing with a Hanks buffer (Beyotime, C0218) and were subsequently fixed using 4% glutaraldehyde (Sigma, G5882). Autophagic vacuoles were quantified using a JEM-1400Plus electron microscope (JEOL, Tokyo, Japan) to examine the cells.

### 2.7. Oil Red O (ORO) Staining

Oil Red O (ORO) (Beyotime, C0157S) is a dye used for lipid staining in histology and biological research. Lipid accumulation in the TM4 cells was assessed using ORO staining. The TM4 cells were washed with Hanks buffer (Beyotime, C0218), then stained with Oil Red O for 20 min. After staining, the cells were washed three times with Hanks buffer and immediately imaged under an Olympus CKX41 microscope (Olympus, Tokyo, Japan).

### 2.8. Measurement of ROS

2′,7′-Dichlorodihydrofluorescein diacetate (DCFH-DA) is a cell-permeable fluorescent probe used to detect the production of reactive oxygen species (ROS) within cells. The DCFH-DA (Beyotime, S0033S) stock solution was diluted 1000 times in DMEM/F12 medium to become the working solution (20 μM). The TM4 cells were washed with Hanks buffer (Beyotime, C0218), then stained with DCFH-DA for 30 min. After staining, the cells were washed three times with Hanks buffer and immediately photographed using a Leica Stellaris 5 confocal laser scanning microscope (Leica, Wetzlar, Germany).

### 2.9. Measurement of Lysosomal pH

LysoSensor Green DND-189 is a fluorescent dye that is primarily used to measure the pH of acidic organelles, such as lysosomes within cells. The LysoSensor Green DND-189 (Meilunbio, MB6043, Dalian, China) stock solution was diluted 1000 times in DMEM/F12 medium to become the working solution (1 μM). The TM4 cells were washed with Hanks buffer (Beyotime, C0218), then stained with LysoSensor Green DND-189 for 1 h. After staining, the cells were washed three times with Hanks buffer and immediately photographed by a Leica Stellaris 5 confocal laser scanning microscope (Leica, Wetzlar, Germany).

### 2.10. Acridine Orange (AO) Staining

Acridine orange (AO) is a cell-permeable nucleic-acid-selective fluorescent dye, mainly used for observing the permeability of lysosomal membranes. The AO (Beyotime, C0233S) stock solution was diluted 1000 times in DMEM/F12 medium to become the working solution (30 μM). The TM4 cells were washed with Hanks buffer (Beyotime, C0218), then stained with AO for 20 min. After staining, the cells were washed three times with Hanks buffer and immediately photographed by a Leica Stellaris 5 confocal laser scanning microscope (Leica, Wetzlar, Germany).

### 2.11. LysoTracker Red Staining

LysoTracker Red is a lysosomal red fluorescent probe that is commonly used for the specific fluorescent staining of lysosomes in live cells. The LysoTracker Red (Beyotime, C0218) stock solution was diluted 10,000 times in DMEM/F12 medium to become the working solution (50 nM). The TM4 cells were washed with Hanks buffer (Beyotime, C0218), then stained with LysoTracker Red for 30 min. After staining, the cells were washed three times with Hanks buffer and immediately photographed by a Leica Stellaris 5 confocal laser scanning microscope (Leica, Wetzlar, Germany).

### 2.12. Apoptosis Assay

Annexin V-FITC (FITC) and Propidium Iodide (PI) are two fluorescent dyes commonly used in apoptosis assays. FITC (BD Pharmingen, 559763, San Diego, CA, USA) and PI (BD Pharmingen, 559763) can effectively distinguish cells in different apoptotic states. The TM4 cells were washed with Hanks buffer (Beyotime, C0218), then stained with annexin FITC and PI for 15 min. After staining, 1 × 10^4^ cells were collected by flow cytometry (BD, Accuri C6) and the proportion of Annexin V-positive cells was analyzed. All groups of cells were examined by flow cytometry within 1 h to ensure the signal strength and data accuracy. The experimental data were evaluated with the help of FlowJo V10 software.

### 2.13. Transfection

A small interfering RNA (siRNA) that targets TFE3 and a control siRNA were provided by Tsingke Biotech (Beijing, China). The siRNA was dissolved in ultrapure water as the stock solution (100 μM). Lipo8000™ Transfection Reagent (Beyotime, C0533) is a high-efficiency nanomaterial-based cell transfection reagent. Prior to transfection, 1 μL of siRNA solution and 1 μL of Lipo8000 transfection reagent were mixed in 1 mL of Opti-MEM medium (Gibco, 31985062, Waltham, MA, USA) for 15 min. Following this, the TM4 cells underwent a 6 h treatment with combined transfection reagents, followed by a transfer to a fresh medium for 24 h to prepare for further experiments.

### 2.14. Immunocytochemical Analysis

Immunofluorescence was conducted following established protocols. Briefly, cells developed to the appropriate density on a circular glass slide situated within a 24-well plate were fixed in 4% paraformaldehyde (Beyotime, P0099) for 30 min, followed by 0.25% Triton X-100 (Beyotime, P0096) permeabilizing for 10 min. Thereafter, cells were blocked with Immunol Staining Blocking Buffer (Beyotime, P0260) for 30 min, and then were incubated at 4 °C overnight in immunostaining buffers with diluted antibodies: rabbit anti-TFEB (1:500, ABclonal, A21657, Wuhan, China), rabbit anti-TFE3 (1:500, ELK Biotechnology, ES7376, Wuhan, China), rabbit anti-LC3 (1:500, CST, 43566S), and rat anti-LAMP-1 (1:250, Invitrogen, LY1C6, Carlsbad, CA, USA). The cells were washed three times using a Western wash buffer (Beyotime, P0023C). Either Donkey Anti-Rabbit IgG (H+L) with Alexa Fluor 555 or Goat Anti-Mouse IgG (H+L) with Alexa Fluor 488 (both from Beyotime, China) were incubated for one hour at 37 °C, diluted 1:200, while shielded from light. The nucleus was stained for 5 min with DAPI staining solution (Beyotime, C1005) to identify the labels, followed by three washes with 5% TBST after the staining procedure. Ultimately, the circular cell-climbing tablets were inverted onto a glass slide and preserved in the Anti-fade Mounting Medium (Solarbio, China) at 4 degrees Celsius while shielded from light. The cell slides were imaged and examined promptly by a Lecia confocal laser scanning microscope (Lecia, Stellaris 5).

### 2.15. Real-Time PCR Analysis

The cells were lysed with Trizol (Beyotime, R0016) and then total RNA was extracted. A total of 1 μg total RNA of each sample was reverse transcribed using a cDNA synthesis kit (ABclonal, RM21400). The RT product was amplified with SYBR Master Mix (ABclonal, RM21203), data were generated by a TFX-96 Real-Time PCR Detection System (Bio-Rad, Hercules, CA, USA), and the relative expression was calculated by the 2^−∆∆CT^ method. The levels of beta-actin gene expression served as the internal standard. The primer sequences for the genes relevant to this study are presented in Table 1.

### 2.16. Western Blot Analysis

The cells were harvested after PBS washing. The cells were lysed with a Western blotting and immunoprecipitation cell lysis buffer (Beyotime, P0013) that contained protease inhibitors (Beyotime, ST506) and incubated on ice for 30 min. The sodium dodecyl sulfate-polyacrylamide gel (Beyotime, P0012A) was then used to separate the protein mixture. Following this, the proteins were moved onto PVDF membranes, which were then incubated with a Blocking Buffer for Western Blotting (Beyotime, P0220) for a duration of 20 min. The membranes were incubated with specific antibodies against LC3 (1:1000, CST, E7X4S), P62 (1:1000, CST, D6M5X), TFEB (1:1000, ABclonal, A21657), TFE3 (1:500, ELK Biotechnology, ES7376), ACTB (1:50,000, ABclonal, AC026), and GAPDH (1:50,000, ABclonal, A19056) at 4 °C overnight. After three TBST washes, the membrane was incubated with HRP-conjugated Goat Anti-Rabbit IgG (Beyotime, A0208) at room temperature for 1 h. The membrane was washed thrice with TBST and then incubated in a chemiluminescent solution for development, and protein bands were obtained. Glyceraldehyde-3-phosphate dehydrogenase (GAPDH) and β-actin (ACTB) were used as internal control proteins.

### 2.17. Statistical Analysis

The results are presented as the mean ± SEM. All quantitative measurements were collected from at least three independent experiments, each carried out in triplicate. The statistical analysis and evaluation of the experimental data were conducted utilizing SPSS 25.0 software. The means of two experimental data sets were compared by unpaired Student’s *t*-tests, while the means of three or more groups were compared by one-way ANOVA, followed by Tukey’s post hoc test. A *p*-value < 0.05 was thought to have statistical significance.

## 3. Results

### 3.1. Lead Exposure Resulted in Reduced Sperm Quality and Increased Autophagy in Mouse Testes

**Figure 1:**
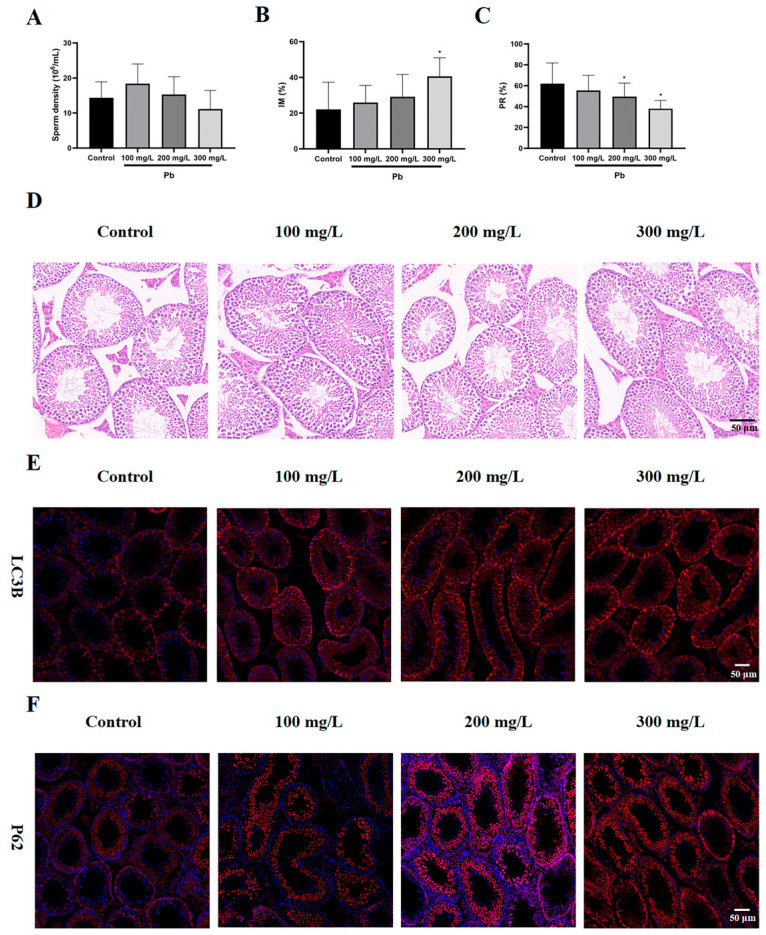
The impact of Pb on sperm movement and the structure of the testicles was investigated. The male mice were exposed to 0, 100, 200, and 300 mg/L Pb for 90 days. (**A**) Sperm density, (**B**) percentage of immotile sperm, and (**C**) percentage of progressive sperm in male mice. (**D**) HE staining of testicular tissue. Immunofluorescence analysis of (**E**) LC3B expression and (**F**) P62 expression. The results are presented as the mean ± SEM and were analyzed via one-way ANOVA. A significance level of * *p* < 0.05 when compared with the control group (*n* = 16).

The sperm density of the three Pb exposure groups (100, 200, and 300 mg/L) were 18.37 × 10^6^/L, 15.3 × 10^6^/L, and 11.15 × 10^6^/L, respectively, and showed no significant reduction when compared with the 14.37 × 10^6^/L of sperm density in the control group (Figure 1A). The proportions of immobile sperm (IM) increased in all three Pb exposure groups (25.94%, 29.13%, and 40.63%), but only the 300 mg/L group exhibited a significantly greater IM proportion compared with the control group (22.06%, *p* < 0.05) (Figure 1B). As shown in Figure 1C, the progressively mobile sperm (PM) in the control group comprised 61.94%, and except for the 100 mg/L group (55.44%), both the 200 mg/L and 300 mg/L groups showed significant decreases in their PMs to 49.5% and 38%, respectively (*p* < 0.05). The structure of the seminiferous tubules showed no obvious variation when comparing the control group with the three experimental groups (Figure 1D). As previously reported, pathological changes in mouse testicles only occurred when the exposure dose reached 1000 mg/L of Pb [24]. Immunofluorescence analysis revealed elevated LC3 and P62 expressions in the testes across all experimental groups, indicating a blockage in the autophagy flow (Figure 1E,F).

### 3.2. Pb Blocked Autophagy Flux in TM4 Cells

**Figure 2:**
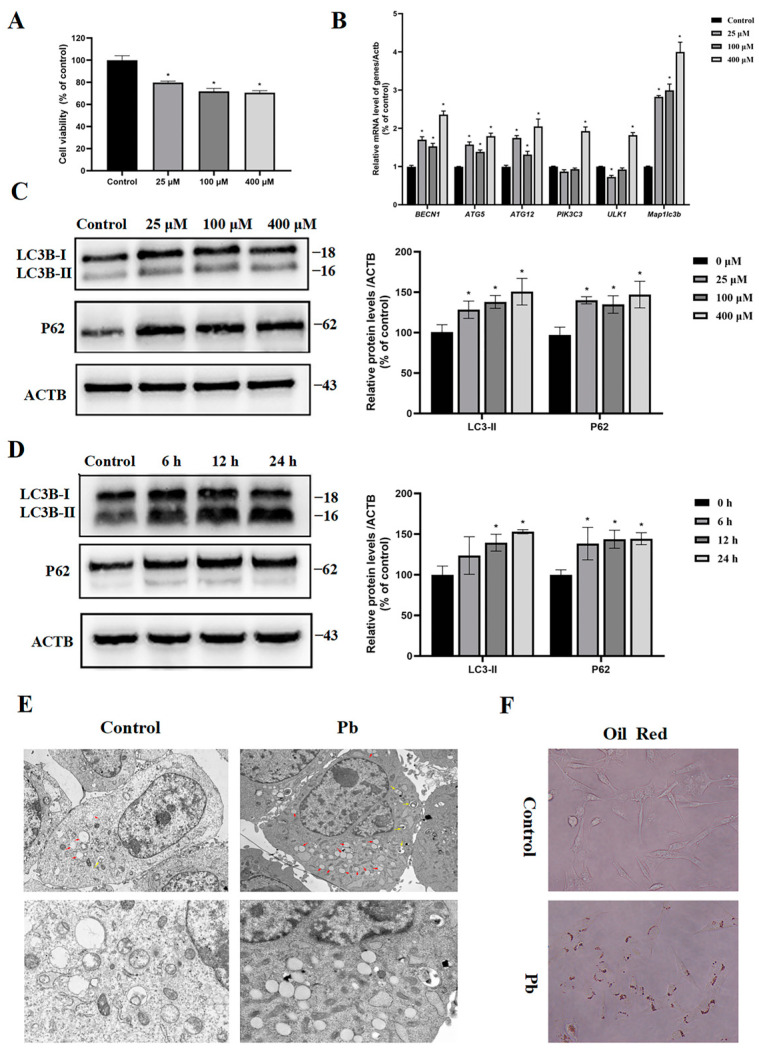
Pb induced the accumulation of autophagosomes and lipid droplets in the TM4 cells. After being exposed to 0, 25, 200, or 400 μM of Pb for 24 h, (**A**) cell viability assessed by an MTT assay, (**B**) mRNA expression levels of autophagy-related genes evaluated by RT-qPCR, (**C**) protein levels of LC3 and P62 subjected to varying concentrations of Pb (from 0 to 400 μM) over a duration of 24 h, and (**D**) 400 μM Pb for different time intervals (from 0 to 24 h). ACTB served as the internal standard. (**E**) TM4 cells were treated with Pb (400 μM) for 24 h, where electron microscopy demonstrated a higher quantity of autophagic vesicles (AVs) and lipid droplets; the lower panels display enlarged sections of the upper panels. The red arrows indicate lipid droplets; yellow arrows indicate autophagosomes. (**F**) Images of oil red staining for cells in the control and Pb (400 μM) treatment groups. The results are presented as the mean ± SEM and were analyzed via one-way ANOVA. A significance level of * *p* < 0.05 when compared with the control group (*n* = 3).

This study examined the 24 h survival rate of the TM4 cells exposed to different Pb concentrations using the MTT assay. The exposure of cells to 25, 100, and 400 μM of Pb led to reductions in the cell viability by approximately 21%, 23%, and 29%, respectively (Figure 2A). The examination of autophagy activation in the TM4 cells implicated that the block of autophagy flux possibly contributed to the observed cellular damage induced by Pb exposure. Of the core genes related to autophagosome formation, including *Becn1*, *Atg5*, *Atg12*, *Ulk1*, *Pik3c3*, and *Map1lc3b*, their mRNA levels exhibited a marked elevation following exposure to Pb, particularly in the 400 μM group, where all the genes showed dramatically increased mRNA expression (Figure 2B). As a common marker for tracking the autophagy flux and measuring the autophagy activity, LC3-II and P62 both had significantly increased protein levels after the Pb treatment, especially the LC3-II level elevated in a time- and dose-dependent manner, which was comparable with the changes in its mRNA expression pattern (Figure 2C,D). Assays for autophagic flux were performed to ascertain whether LC3-II accumulation was due to autophagy induction or a subsequent process blockage. The increased P62 levels after Pb treatment implied the inhibition of autophagic degradation (Figure 2C,D). In addition, transmission electron microscopy revealed a significant rise in the quantity of autophagosomes and lipid droplets within the TM4 cells (Figure 2E) and the oil red test also confirmed the increased lipid accumulation in the TM4 cells in the Pb-treated group (Figure 2F). The results mentioned above indicate that Pb-induced autophagy may manage intracellular lipid concentrations by degrading the lipid droplets, a process referred to as lipophagy, in TM4 cells.

### 3.3. Preventing the Fusion of Autophagosomes with Lysosomes Reduced Pb-Induced Cell Damage

**Figure 3:**
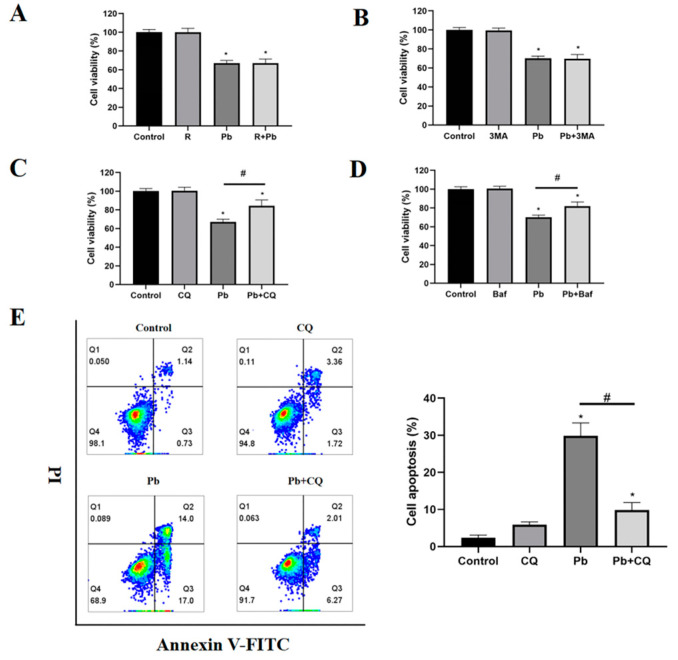

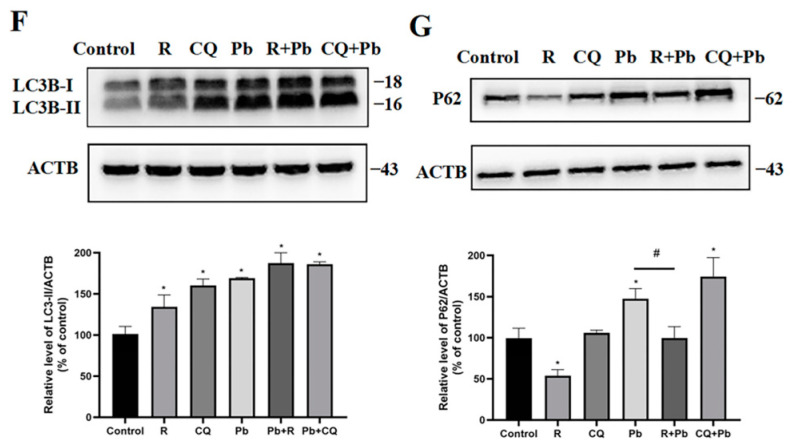
Chloroquine reduces Pb-induced cytotoxicity by inhibiting the autophagosome–lysosome fusion in the TM4 cells. After the treatment with Pb (400 μM) for 24 h, the MTT assay was used to access the cell viability both in the absence and presence of (**A**) rapamycin (R, 200 nM), (**B**) 3-MA (2 mM), (**C**) bafilomycin A1 (Baf, 10 nM), and (**D**) chloroquine (CQ, 20 μM). After the treatment with Pb (400μM) for 48 h, flow cytometry was used to detect the (**E**) apoptosis in the TM4 cells with or without chloroquine (CQ, 20 μM). An analysis that involved immunoblotting and quantification was conducted for (**F**) LC3-II and (**G**) P62 after treating TM4 cells with Pb (400 μM) for 24 h, either with or without the presence of rapamycin (R, 200 nM) or chloroquine (CQ, 20 μM). ACTB served as the internal standard. The results are presented as the mean ± SEM and were analyzed via one-way ANOVA. * *p* < 0.05 when compared with the control group; # *p* < 0.05 when compared with the Pb (400 μM) group (*n* = 3).

To assess autophagy’s role in Pb-induced cell death, rapamycin, 3MA, CQ, and Baf were used to treat the cells exposed to Pb to modulate the autophagy levels. 3MA and rapamycin act as inhibitors and activators respectively in the initiation stage of autophagy; both CQ and Baf had the ability to disrupt lysosomal acidification and prevent the merging of autophagosomes with lysosomes. Neither 3-MA nor rapamycin influenced the cytotoxic effects induced by Pb (Figure 3A,B). These results suggest that both the inhibition and promotion of autophagosome formation failed to safeguard the cells against Pb-induced cell death. In contrast, CQ and Baf significantly increased the survival of the Pb-treated cells, indicating that the Pb-induced damage was restrained by controlling the autophagy maturation stage (Figure 3C,D). To further validate this finding, the impact of CQ on the apoptosis of the TM4 cells exposed to Pb was subsequently assessed. Since our previous experimental results show that there was no obvious apoptosis after 24 h of Pb treatment, we extended the treatment time to 48 h. Consistent with our assumptions, the CQ treatment significantly reduced the apoptosis induced by Pb (Figure 3E). Immunoblotting results confirmed that rapamycin reduced the p62 expression after Pb exposure, indicating that rapamycin restored the autophagic flux (Figure 3G). The presence of CQ did not significantly enhance LC3-II accumulation induced by Pb, supporting the hypothesis that Pb disrupts autophagosome–lysosome fusion (Figure 3F).

### 3.4. The Autophagosomes–Lysosomes Fusion and Lysosomal Function in the TM4 Cells Were Disrupted by Pb

**Figure 4:**
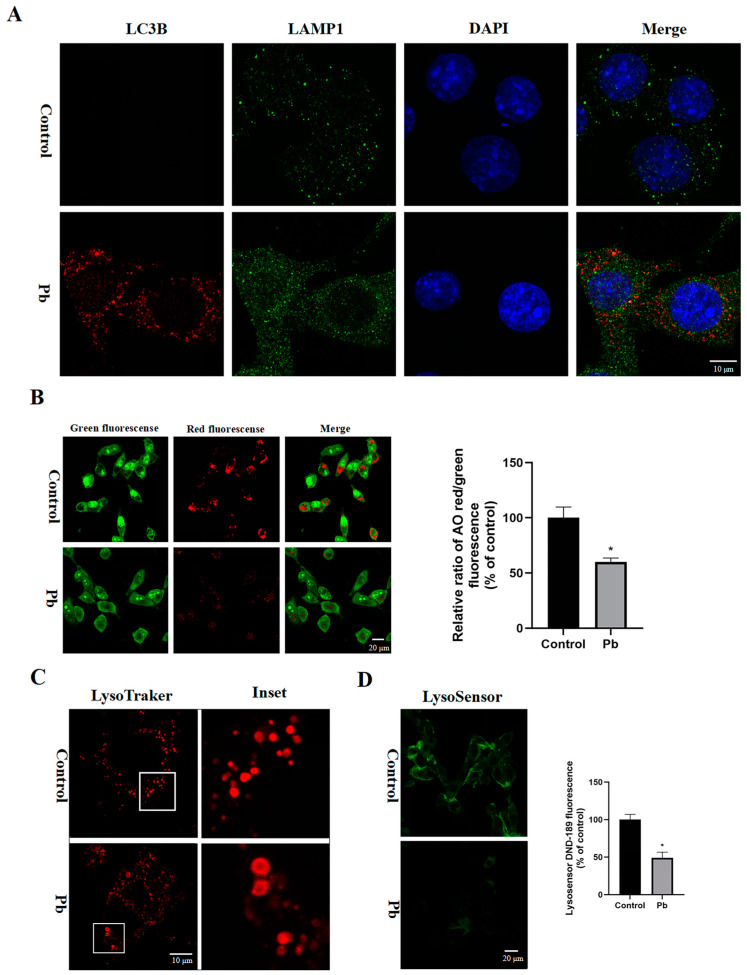
Pb disrupted the autophagosome–lysosome fusion and lysosomal function. The TM4 cells were treated with Pb (400 μM) for 24 h. (**A**) Immunofluorescence staining was used to label LC3 and LAMP1. (**B**) Acridine orange fluorescence was employed to study the LMP (lysosomal membrane permeabilization) through confocal microscopy. (**C**) LysoTracker Red fluorescence served to assess the morphology of lysosomes using confocal microscopy. (**D**) LysoSensor Green DND-189 fluorescence served to assess the lysosome pH using confocal microscopy. The results are presented as the mean ± SEM and were analyzed via one-way ANOVA. A significance level of * *p* < 0.05 when compared with the control group (*n* = 3).

The autophagosomes, which contain cellular waste material, must first fuse with lysosomes in order to be degraded and recycled by the cell. The immunostaining for LC3 and LAMP1 was performed to assess the process of autophagosome–lysosome fusion under Pb exposure; as shown in Figure 4A, the LC3-positive autophagosome (red) were not co-localized well with the LAMP1-positive lysosome (green). Given that red fluorescence means acridine orange (AO) accumulation in lysosomes, AO staining was performed to determine whether LMP (lysosomal membrane permeabilization) is affected by Pb exposure, and the cell images with significantly decreased red fluorescence may implicate the reduction of AO accumulation in lysosomes (Figure 4B). In addition, by using LysoTracker Red staining to trace the morphological change of lysosomes, an increased number of lysosomes that swelled into large vacuoles was found (Figure 4C), which implicates that the Pb caused lysosome morphological changes and impaired the function of the lysosomes. Sequentially, the lysosome pH was tested and an increased pH value of lysosomes in the Pb-treated group was observed that also indicated the impaired function of lysosomes (Figure 4D). Based on these data, the Pb blocked the merging of autophagosomes with autolysosomes, disrupted lysosomal function, and consequently resulted in the buildup of LC3 positive autophagosomes.

### 3.5. Pb Treatment Caused Nuclear Translocation of TFE3 Protein

**Figure 5:**
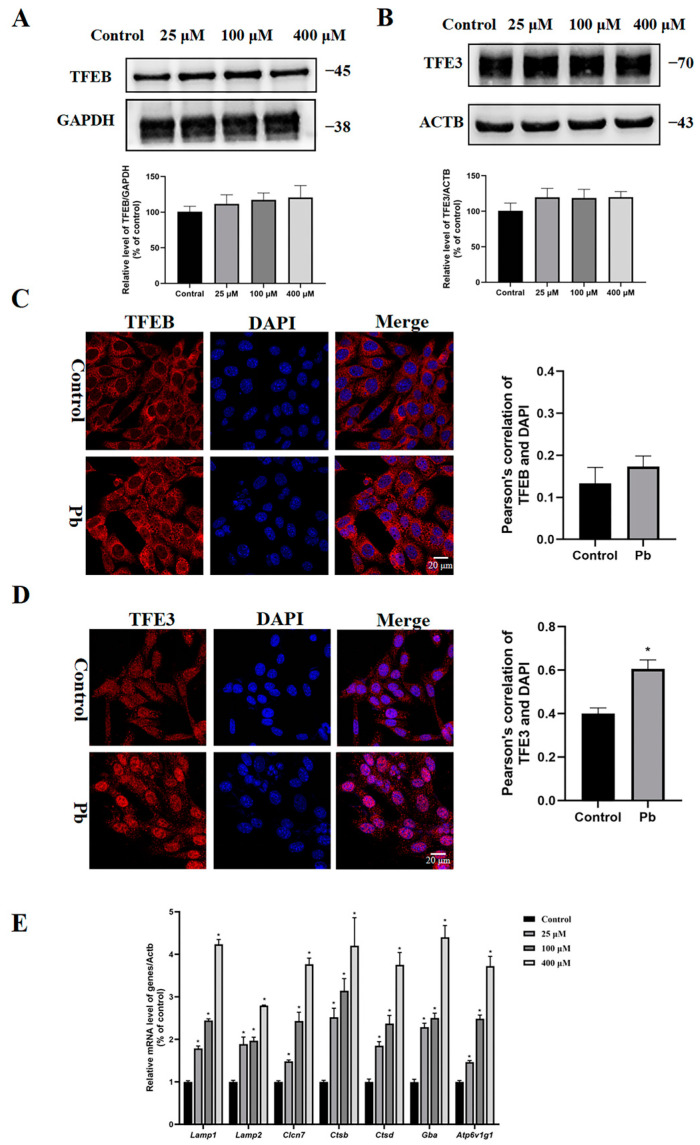
Pb induced the nuclear translocation of TFE3 in the TM4 cells. After exposure to 0, 25, 100, or 400 μM of Pb for 24 h, immunoblotting and quantification were used to assess the protein levels of (**A**) TFEB and (**B**) TFE3. GAPDH and ACTB served as the internal standard. Immunofluorescence analysis of the 400 μM Pb group for 24 h using the (**C**) anti-TFEB antibody and (**D**) anti-TFE3 antibody. (**E**) mRNA expression levels of the TFE3 target genes, including *Lamp1*, *Lamp2*, *Clcn7*, Ctsb, *Ctcd*, *Gba*, and *Atp6v1g1*. The results are presented as the mean ± SEM and were analyzed via one-way ANOVA. A significance level of * *p* < 0.05 when compared with control group (*n* = 3).

Since the MiT/TFE family members TFEB and TFE3 can interact with CLEAR elements and then regulate the expression of relative autophagic and lysosomal genes, we further explored whether TFEB and TFE3 were involved and their potential roles in the Pb-induced autophagy in TM4 cells. Immunoblot analysis indicated that the TFEB and TFE3 expression levels remained unchanged in TM4 cells exposed to Pb (Figure 5A,B), but the TFE3 protein translocation into the nucleus was observed after the Pb exposure (Figure 5C,D). As expected, treatment with Pb resulted in a notable enhancement of mRNA levels for genes associated with lysosomal biogenesis, including lysosomal transmembrane proteins (*Lamp1*, *Lamp2*, and *Clcn7*), lysosomalhydrolases (*Ctsb*, *Ctsd*, and *Gba*), and lysosomal V-ATPase (*Atp6v1g1*) (Figure 5E). Taken together, our results imply that the elevated expression of TFE3 response genes was very likely associated with Pb-induced TFE3 protein nuclear translocation.

### 3.6. TFE3 Mediated Pb-Induced Autophagy in TM4 Cells

**Figure 6:**
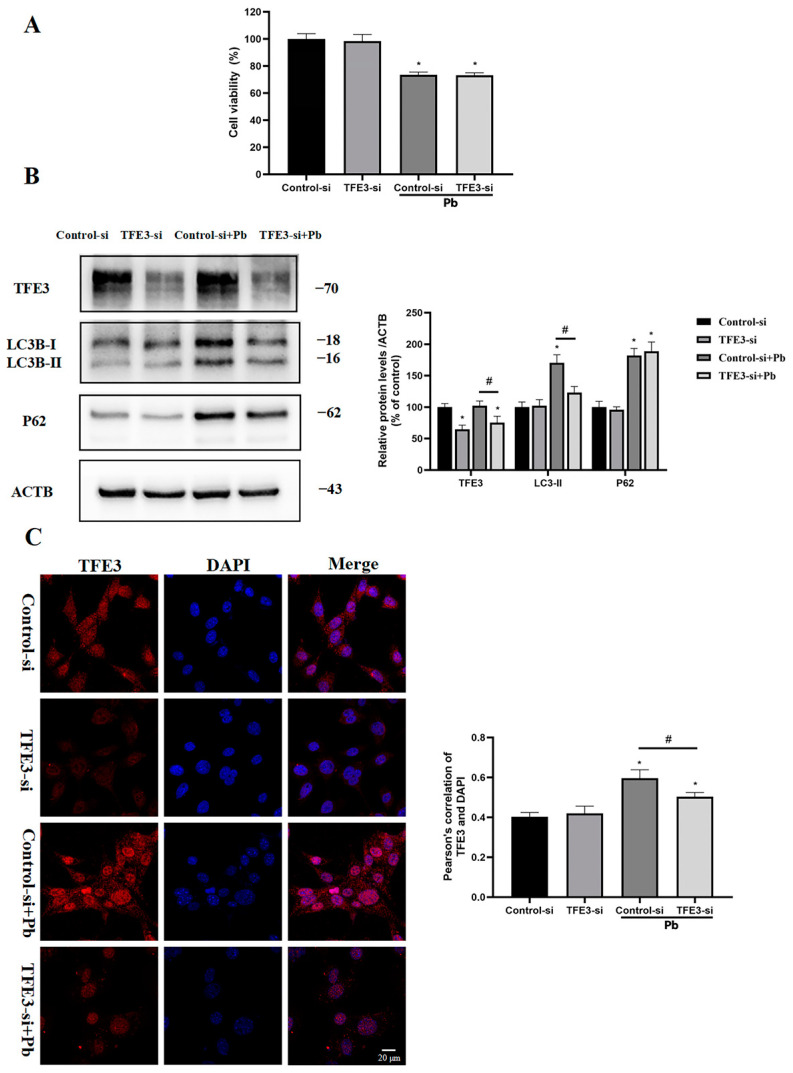
Pb activated the TFE3-mediated autophagy pathway. The TM4 cells were transfected with 100 nM TFE3 siRNA or control siRNA and then treated with 400 μM Pb for 24 h. (**A**) Viability of TM4 cells. (**B**) The immunoblot and quantification analysis of TFE3, LC3-II, and P62 in TM4 cells. ACTB served as the internal standard. (**C**) The immunofluorescence analysis was conducted on the TM4 cells using anti-TFE3 antibody and DAPI. The results are presented as the mean ± SEM and were analyzed via one-way ANOVA. * *p* < 0.05 when compared with the control group; # *p* < 0.05 when compared with the Pb (400 μM) group (*n* = 3).

To evaluate the TFE3 function of the TFE3 in the Pb-triggered autophagy, RNAi-mediated silencing of the TFE3 was performed. Analysis through immunoblotting revealed that the administration of TFE3 siRNA decreased the levels of TFE3 and LC3-II following the exposure to Pb, while it did not influence the expression of p62 (Figure 6B). The immunofluorescence analysis indicated that TFE3 siRNA notably diminished the nuclear translocation of TFE3 (Figure 6C). Our results demonstrated that TFE3 siRNA reduced the level of Pb-induced autophagy. However, TFE3 knockdown did not restore the autophagic flux in Pb-induced cells, nor did it restore the cell viability (Figure 6A). In summary, the TFE3 played a role in the Pb-induced autophagosome formation phase.

### 3.7. Inhibition of ROS Partially Restored Autophagic Flux and Reduced Lipid Droplet Accumulation

**Figure 7:**
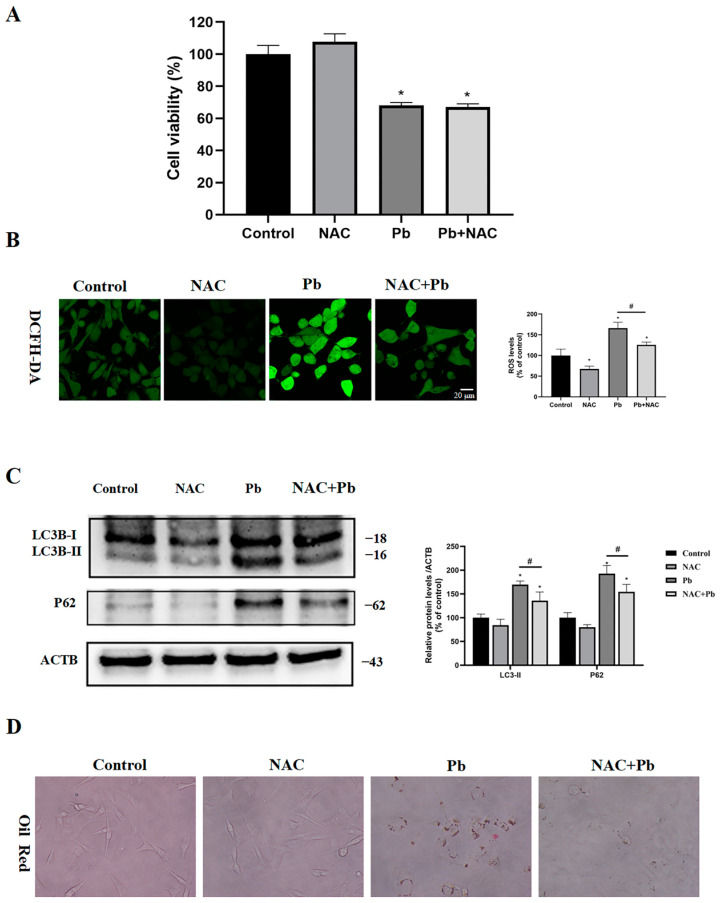
In the TM4 cells, the ROS acted as the upstream signaling molecule that triggered the Pb-induced autophagy. The TM4 cells were treated with 400 μM Pb, with or without NAC (2 mM), for 24 h. (**A**) Viability of the TM4 cells. (**B**) ROS levels of the TM4 cells. (**C**) A representative immunoblot and quantification analysis of LC3-II and P62 in the TM4 cells were performed. ACTB served as the internal standard. (**D**) Microscope image of the TM4 cells stained with oil red. The results are presented as the mean ± SEM and were evaluated using one-way ANOVA. * *p* < 0.05 when compared with the control group; # *p* < 0.05 when compared with the Pb (400 μM) group (*n* = 3).

Considering the essential role of ROS in autophagy, it is vital to examine its involvement in autophagy and the cytotoxic effects triggered by Pb. As shown in Figure 7, the antioxidant NAC had no protective effect on the viability of cells exposed to Pb (Figure 7A), though the elevated levels of ROS after Pb exposure was significantly mitigated by the NAC treatment (Figure 7B). These results suggest that ROS may not be a key contributor in Pb-induced toxicity. Concomitantly, LC3-II and P62 protein levels were found to be significantly reduced by the NAC treatment (Figure 7C), which indicates that ROS played a critical role in autophagy. Notably, cells in the NAC- and Pb-treated groups showed only a small accumulation of lipid droplets (Figure 7D), suggesting that restoring autophagic flux can effectively decompose lipid droplets.

## 4. Discussion

Pb is a widespread environmental pollutant known to cause male reproductive tox-icity. Pb-induced toxic effects on Sertoli cells, one of the most important functional cells in testes, have been reported [25,26]; however, whether autophagy plays a role and the underlying mechanisms remain unclear. Autophagy, a process of membrane trafficking that has been conserved throughout evolution, is essential for preserving intracellular metabolism and energy balance by breaking down damaged organelles and cytoplasmic proteins [27]. In many instances, autophagy is regarded as a protective mechanism for cells. However, when autophagy is overactivated or dysfunctional, causing damage to organelles, it can be harmful to cells. Recently, multiple research investigations indicated a link between autophagy impairment and the decline in Sertoli cell functionality. The autophagy pathway is essential for maintaining Sertoli cell polarity and enhancing male fertility by removing SCIN and HDAC6, which negatively impact F-actin cytoskeleton organization [28]. Apoptotic cells are cleared from the system through the phagocytosis and autophagy of Sertoli cells, providing a normal environment for sperm maturation [29]. An investigation into the expression profiles of microRNAs and their functional networks within mouse testes following Pb exposure showed that the pathways that are associated with lysosomes and autophagy were the most affected pathways [30]. An electron microscopic observation of Pb-exposed rats showed that the lysosome content increased in Sertoli cells [13]. Clarifying the mechanisms of autophagy and lysosomal pathways in Pb-induced Sertoli cell death is crucial.

This research demonstrated that Pb toxicity is associated with alterations in the autophagy levels. In fact, we found that some autophagy-related key genes, such as *Becn1*, *Atg5*, *Atg12*, *Ulk1*, *Pik3c3*, and *Map1lc3b*, were upregulated after the Pb exposure, and the Pb-induced increase in the LC3-II and P62 levels suggests an obstruction of autophagic degradation within lysosomes. In the meantime, immunofluorescence also showed low levels of colocalization of LC3 and Lamp1, which implies that the autophagosome–lysosome merging was inhibited. Our research showed that Pb caused lysosomal membrane permeabilization, lysosome expansion into large vacuoles, and the elevation of pH in lysosome. The results support that Pb disrupts autophagic flow in TM4 cells by impeding autophagosome–lysosome fusion and reducing lysosomal activity. The function of autophagy concerning lead toxicity continues to be a debated issue. Earlier research has indicated that cell death through autophagy is associated with the impairment of learning and memory that results from Pb exposure [31,32]. In contrast, autophagy functions as a protective factor in the death of osteoblasts following Pb treatment [33]. Our research indicates that blocking the formation of autophagosomes failed to safeguard the cells from injury caused by Pb, suggesting it was not autophagic cell death. In addition, restored autophagic flux also failed to improve the cell survival. Surprisingly, the inhibition of autophagosome–lysosome fusion significantly improved the cell viability, suggesting that targeting the autophagy maturation stage could be a potential strategy for mitigating the cytotoxic effects of Pb.

With regard to TFE3, this is a member of the MiT/TFE helix–loop–helix subfamily that is linked to autophagy and lysosomal function [34]. Notably, the MiT/TFE family exists in an inactive form in the cytoplasm and enters the nucleus after activation. Nuclear translocation of the MiT/TFE family modulates lysosomal function and crucial phases of autophagy, including autophagosome formation, their fusion with lysosomes, and substrate degradation [35]. The capacity of TFE3 to carry out two contrasting cellular outcomes is influenced by the type of cell and the specific stimulus. For example, TFE3 overexpression maintains the lysosome–mitochondria axis and subsequently protected neurons from cadmium-induced apoptosis [36]. When autophagy is harmful to cells, the activation of TFE3 might trigger excessive autophagy and possibly cause cell death [37]. Given the fact that the nuclear translocation of TFE3 may upregulate the transcription of its target genes, TFE3 is likely activated in the compensatory role after Pb-induced impairment of autophagic flux. The use of TFE3 siRNA to inhibit autophagy activation indicates TFE3’s role in regulating the prolonged phase of autophagy. However, the knockdown of TFE3 had no effect on cell viability, demonstrating that the cytotoxicity by Pb is not restrained by the aggregation of autophagosomes. Further investigation is required to determine whether TFE3 overexpression protects against Pb-induced injury.

Reactive oxygen species (ROS) are involved in cellular processes like apoptosis, ne-crosis, and autophagy. The generation of ROS due to drug metabolism and environmental contaminants can lead to significant harm to macromolecules and organelles within cells. In some reports, pretreatment with N-acetylcysteine (NAC) demonstrates a notable beneficial impact on the ROS generated by Pb exposure, as well as on DNA damage and programmed cell death [38]. After the Pb treatment, the ROS levels in the TM4 cells were significantly increased, which was inhibited by NAC. We found that the antioxidant NAC restored the autophagic flux and significantly reduced the accumulation of lipid droplets. In fact, therapeutic approaches to reduce lipid droplet accumulation by increasing autophagy have been used in hepatic steatosis. TFE3 overexpression alleviates hepatic steatosis by activating autophagy and PGC-1α-mediated fatty acid metabolism [39]. These data indicate that Pb induces multiple signaling pathways for autophagy in TM4 cells. Indeed, the suppression of ROS does not safeguard TM4 cells against cell death induced by Pb, indicating that oxidative stress may not be a critical target of lead cytotoxicity.

Overall, our study filled a gap in comprehending the fundamental mechanisms as-sociated with Pb-induced reproductive toxicity, offering insights into the function of au-tophagy in the molecular events triggered by Pb. Notably, blocking autophagosome–lysosome fusion could present a novel approach for addressing reproductive toxicity caused by Pb exposure, such as mitochondrial generation and energy metabolism.

## Figures and Tables

**Table 1:** Primer used in the current study.

	Forward Sequence (5′–3′)	Reverse Sequence (5′–3′)
*Becn1*	ATGGAGGGGTCTAAGGCGTC	TGGGCTGTGGTAAGTAATGGA
*Atg5*	TGTGCTTCGAGATGTGTGGTT	ACCAACGTCAAATAGCTGACTC
*Atg12*	CGGAAGATTCAGAGGTTGTGCT	CAGCCTTCAGCAGGATGTCAA
*Ulk1*	AAGTTCGAGTTCTCTCGCAAG	ACCTCCAGGTCGTGCTTCT
*Pik3c3*	GGGCTATACCAAGAGACATGC	CGCCTTGTAGGATGTTCTGACT
*Map1lc3b*	TTATAGAGCGATACAAGGGGGAG	CGCCGTCTGATTATCTTGATGAG
*Lamp1*	CAGCACTCTTTGAGGTGAAAAAC	CCATTCGCAGTCTCGTAGGTG
*Lamp2*	TGTATTTGGCTAATGGCTCAGC	TATGGGCACAAGGAAGTTGTC
*Clcn7*	GACAACAGCGAGAATCAGCTC	CCAATGAGGGCACAGATAACC
*Ctsb*	CAGGCTGGACGCAACTTCTAC	TCACCGAACGCAACCCTTC
*Ctsd*	CGATTATCAGAATCCCTCTGCG	GGTCTTAGGCGATGACTGCAT
*Gba*	GTATGGCCTAAGATTCTGGGC	CTAGGTCACGGGAAATGAAGTC
*Atp6v1g1*	CCCAGGCTGAAATTGAACAGT	TTCTGGAGGACGGTCATCTTC

## Data Availability

The supporting data for the findings of this research can be obtained from the corresponding author upon a reasonable request.
